# Technical review of endoscopic ultrasound‐guided drainage/anastomosis and trans‐endosonographically created route procedures for the treatment of pancreatic diseases

**DOI:** 10.1002/deo2.393

**Published:** 2024-06-07

**Authors:** Ko Tomishima, Hiroyuki Isayama, Akinori Suzuki, Shigeto Ishii, Sho Takahashi, Toshio Fujisawa

**Affiliations:** ^1^ Department of Gastroenterology Graduate School of Medicine Juntendo University Tokyo Japan

**Keywords:** pancreatic ductal stricture, EUS‐D/A, T‐DAS, interventional EUS, EUS‐PDD

## Abstract

Endoscopic ultrasound (EUS)‐guided pancreatic duct drainage includes two procedures: EUS‐guided drainage/anastomosis (EUS‐D/A) and trans‐papillary drainage with EUS‐assisted pancreatic rendezvous. EUS‐guided pancreatogastrostomy is the most common EUS‐D/A procedure and is recommended as a salvage procedure in cases in which endoscopic retrograde cholangiopancreatography fails or is difficult. However, initial EUS‐D/A is performed in patients with surgically altered anatomy at our institution. It is one of the most difficult interventional EUS procedures and has a high incidence of adverse events. The technical difficulties differ according to etiology, and the incidence of adverse events varies between initial EUS‐D/A and subsequent trans‐endosonographically/EUS‐guided created route procedures. Hence, it is important to meticulously prepare a procedure based on the patient's condition and the available devices. The technical difficulties in EUS‐D/A include: (1) determination of the puncture point, (2) selection of a puncture needle and guidewire, (3) guidewire manipulation, and (4) dilation of the puncture route and stenting. Proper technical procedures are important to increase the success rate and reduce the incidence and severity of adverse events. The complexity of EUS‐D/A is also contingent on the severity of pancreatic fibrosis and stricture. In post‐pancreatectomy cases, determination of the puncture site is important for success because of the remnant pancreas. Trans‐endosonographically/EUS‐guided created route procedures following initial EUS‐D/A are also important for achieving the treatment goal. This article focuses on effective strategies for initial EUS‐D/A, based on the etiology and condition of the pancreas. We mainly discuss EUS‐D/A, including its indications, techniques, and success‐enhancing strategies.

## INTRODUCTION

Endoscopic ultrasound‐guided pancreatic duct drainage (EUS‐PDD) is performed to treat recurrent pancreatitis in patients with endoscopic retrograde cholangiopancreatography (ERCP) failure or pancreato‐enteric anastomotic stricture (PEAS).^1^, ^2^ It is an effective salvage technique when pancreatic access by ERCP or ERCP with balloon‐assisted endoscopy (BAE‐ERCP) fails or is difficult.^3^, ^4^, ^5^ There are two types of EUS‐PDD: EUS‐guided drainage/anastomosis (EUS‐D/A) and transpapillary/anastomosis pancreatic drainage with rendezvous technique, which was first reported in 2002.^6^ EUS‐PDD as EUS‐D/A is divided into EUS‐guided pancreaticogastrostomy (EUS‐PGS), EUS‐guided pancreatojejunostomy, and EUS‐guided pancreatoduodenostomy, depending on the approached organ. EUS‐assisted rendezvous technique is performed in cases of failed cannulation to the papilla/anastomosis using a duodenoscope and BAE. However, the success rate of pancreatic duct drainage with the EUS‐assisted rendezvous technique is low because of the difficulty of guidewire manipulation.^7^ Finally, transluminal drainage/anastomosis stent (T‐DAS) can be placed through the endosonographically/EUS‐guided created route (ESCR). T‐DAS aims to retain the ESCR and differs from conventional stents, which are placed at the stricture/papilla. T‐DAS and ESCR were proposed by the Subcommittee of Terminology of Interventional EUS (I‐EUS) of the Japanese Gastroenterological Endoscopy Society.

EUS‐D/A is one of the most challenging types of I‐EUS in terms of low technical success and high adverse event (AE) rates. In one study, 27 EUS‐D/A cases were required to reach an 80‐min procedure duration, and the duration plateaued after 40 cases.^8^ The technical success rate was 84% in 56 cases and the AE rate was 23.2%. The authors concluded that EUS‐D/A is one of the most difficult therapeutic endosonographic procedures to learn. According to one review, EUS‐D/A has a high rate of AEs (21.8%) of pancreatitis, pancreatic leak, pancreatic pseudocyst, peritonitis, hemorrhage, and perforation; the technical success rate is low (78.7%).^7^ Because serious complications can occur during EUS‐D/A, it is essential to be familiar with the technique and the device. The lack of dedicated devices for EUS‐D/A, scope instability, and the stiff and fibrotic pancreatic parenchyma cause difficulties. In patients with a surgically altered anatomy (SAA), the puncture site is limited by the size of the remnant stomach or pancreas. It is important to minimize the rates of serious complications.

Managing postoperative complications is important and the minimum requirement for performing EUS‐PDD. In addition, after the maturation of the ESCR, various procedures can be performed in the pancreatic duct. Strategies including trans‐ESCR should be considered to achieve the treatment goals. Here we provide tips for EUS‐D/A.

### Indications for EUS‐PDD

There are two indications for EUS‐PDD: symptomatic cases with a main pancreatic duct (MPD) requiring drainage or the creation of an approach route to perform an intraductal procedure, and cases in which access to the MPD failed or was difficult.^9^, ^10^, ^11^ Common causes of such complications include MPD stricture, PEAS, divisum, impacted stones, duodenal stenosis, and disconnected pancreatic duct syndrome (DPDS).^3^ Failed pancreatic access can be caused by anatomical status (inaccessible major/minor papilla or anastomotic site) or procedural issues (cannulation failure and failure to pass the MPD). Patients with recurrent pancreatitis, symptomatic chronic pancreatitis (CP), pseudocysts, pancreatic juice leakage, and fistulas may benefit from EUS‐PDD. Contraindications include patient‐related factors (coagulopathy, massive ascites, and severe sickness) and anatomical factors (non‐dilated MPD, long distance between the gastrointestinal wall and MPD, and the presence of unavoidable intervening large vessels).

### Algorithm for pancreatic duct obstruction/leakage used at our institution

**FIGURE 1:**
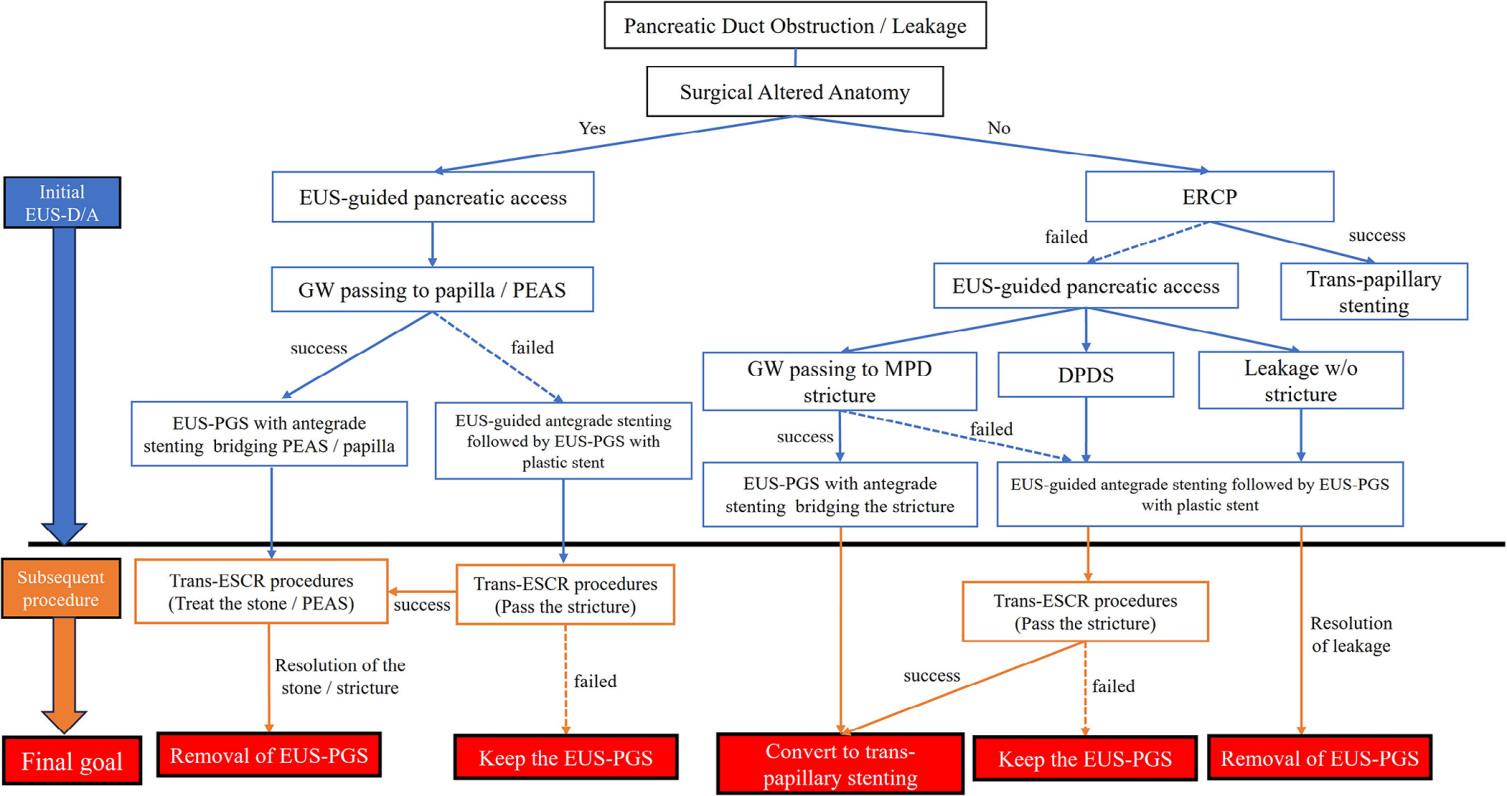
Pancreatic duct obstruction and leakage algorithm used at our institution.

Endoscopic retrograde pancreatography by duodenoscopy or colonoscopy has a low technical success rate (8%),^12^ compared to 70.7% for BAE‐ERCP^13^ in post‐Whipple patients. A combination of BAE‐ERCP and EUS‐PDD can achieve high technical and clinical success rates for pancreatic diseases in patients with SAA. At our institution, we prefer EUS‐D/A over the rendezvous method, because it is less complex and time‐consuming. EUS‐assisted rendezvous technique can be performed for patients with normal anatomy in whom the stenosis can be smoothly passed from the puncture, but EUS‐D/A is often performed for ERCP failure because of the difficulty passing the stenosis after the puncture. Peroral pancreatoscopy enables assessment of postoperative recurrence following endoscopic intervention via T‐DAS placed at EUS‐D/A (trans‐ESCR procedure) with an acceptable AE rate.^14^ A reliable diagnosis alters the management of EUS‐PGS. For example, if the PEAS or MPD stricture is a neoplastic stenosis, EUS‐PGS can be retained, and if a benign stricture is released, it can be closed. In other words, the final goal of the management of EUS‐PGS varies depending on the etiology, so a reliable diagnosis (i.e., stricture diagnosed via trans‐ESCR) is mandatory (Figure 1).

### Etiologies requiring EUS‐PDD and technical and clinical success and AE rates

Some etiologies require EUS‐PDD (e.g., PEAS, CP, gallstone pancreatitis, intraductal papillary mucinous neoplasm, acute pancreatitis, sphincter of Oddi dysfunction, pancreatic cancer, divisum, and tumors; Table 1). In our literature review, the numbers of cases with these etiologies were 165, 104, and 30, respectively, and the technical and clinical success rates did not significantly differ between PAS and CP (85.5% and 83.7%, *p* = 0.8 / 82.7%, and 81.3%, *p* = 1.0; Table 2). There is no standard definition of AEs in EUS‐PDD, and the onset time is inconsistent among studies. Lexicon by Cotton PB and the Clavien‐Dindo score have been modified and are used to evaluate EUS‐PDD (12–14). Standard definitions are warranted in EUS‐PDD, such as the Tokyo criteria for trans‐papillary biliary stenting. We classified AEs according to onset time into procedure‐related AEs (≤1 month) and late AEs (>1 month). The rates of procedure‐related AEs were 0–38% and included abdominal pain, pancreatitis, pancreatic leakage, peripancreatic fluid collection, peritonitis, stent migration, bleeding, aneurysm, jejunal ulcer, and perforation (Table 3). Retrieving a migrated plastic stent from the pancreatic duct is challenging, even when the distal end of the stent is proximal to the puncture point.^15^ For this reason, we recommended EUS‐PGS with antegrade stenting to bridge the stricture using a double pigtail stent (referred to as ring drainage). To facilitate early detection of AEs, routine plane CT is performed, even in the absence of symptoms.^16^

**TABLE 1:** Etiologies requiring endoscopic ultrasound‐pancreatic duct drainage (EUS‐PDD) and technical and clinical success and adverse event rates.

Author	Etiology	*n*	Technical success	Clinical success	Procedure‐related AEs (≦ 1 month)	Late AE (>1 month)	Median follow‐ up	Definition of AEs	Details of others
			rate (%)	rate (%)	Cases (%) / details	Cases (%) / details	months		
Kahaleh et al.	PEAS	7	5 (71.4)	NA	2/13 (15.3)/1 bleeding and 1 perforation	NA	NA	NA	2 complications resulting from gallstone pancreatitis 1 IPMN
	CP	3	3 (100)	NA					
	Others	3	3 (100)	NA					
Brauer et al.	CP	6	4 (66.6)	4/8 (50)	None; 0/8 (0)	NA	NA	NA	2 acute pancreatitis
	Others	2	0 (0)						
Barkay et al.	PEAS	9	2 (22.2)	NA	2/20 (10)/1 pancreatitis and 1 peripancreatic abscess	NA	NA	Cotton et al.	4 SOD and 2 acute recurrent pancreatitis
	CP	6	3 (50)						
	Others	6	5 (83.3)						
Ergun et al.	PEAS	8	7 (87.5)	NA	2/20 (10)/1 bleeding and 1 perigastric collection	9/18 (50)/Seven stent occlusions and two migrations	37 (3–120)	NA	
	CP	12	11 (91.7)						
Kurihara et al.	PEAS	14	12 (85.7)	12/12 (100)	1/17 (5.9)/1 aneurysm and pseudocyst	NA	NA	NA	2 acute pancreatitis
	CP	1	1 (100)	1/1 (100)					
	Others	2	2 (100)	2/2 (100)					
Fujii et al.	PEAS	27	20 (74.1)	11/20 (55)	3/44 (6.8)/1 peripancreatic abscess, 1 pancreatitis, and 1 guidewire sharing	NA	NA	NA	NA
	CP	17	11 (64.7)	NA					
Chen et al.	PEAS	40	37 (92.5)	32/40 (80)	15/40 (35)/13 abdominal pain, one intra‐abdominal absess, and 1 jejunal ulcer	NA	NA	Cotton et al.	NA
Uchida et al.	PEAS	6	4 (66.6)	4/4 (100)	2/15 (13.3)/2 peritonitis	2/15 (13.3)/1 bleeding and 1 stent migration	6.7	NA	7 pancreatic cancers
	CP	2	2 (100)	2/2 (100)					
	Others	7	7 (100)	6/7 (85.7)					
Matsunami et al.	PEAS	21	21 (100)	21/21 (100)	7/30 (23)/5 pain, 1 pancreatitis, and 1 severe bleeding	6/25 (24)/6 stent dislodgements	23 (6–44)	NA	1 divisum and 2 cancers
	CP	6	6 (100)	6/6 (100)					
	Others	3	3 (100)	3/3 (100)					
Krafft et al.	PEAS	2	2 (100)	17/22 (77)	4/28 (14.2)/1 luminal hemorrhage, 1 microperforation, and two leakages of pancreatic secretion	None; 0/15 cases (0)	4.5 (IQR 3–7.75)	Cotton et al.	NA
	CP	26	23 (88)						
Rudler et al.	PEAS	21	21 (100)	15/20 (75)	8/21 cases (38)/ 1 pancreatitis, 6 pain, and 1 postprocedure fluid collection	5/21 (24)/1 migration and 4 blocked stents	24	Clavien et al.	NA
	CP	22	20 (90.9)	14/20 (70)	7/22 cases (32)/1 bleeding, 1 pancreatitis, 3 pain, and 2 stent migrations	7/22 (31)/5 migrations and 2 blocked stents	10		
Suzuki et al.	PEAS	10	10 (100)	10/10 (100)	4/20 (20)/3 pancreatic juice leakages, 1 mild	3/20 (15)/2 stent migrations and 1 mild bleeding	21 (IQR 13–30)	Cotton et al.	3 tumors, 3 DPDS, and 1 divisum
	CP	3	3 (100)	3/3 (100)	pancreatitis				
	Others	7	6 (85.7)	6/6 (100)					
Total		299	254 (84.9)	148/177 (83.6)					

Abbreviations: AEs, adverse events; CP, chronic pancreatitis; DPDS, disconnected pancreatic duct syndrome; IPMN, intraductal papillary mucinous neoplasm; IQR, interquartile range; NA, not applicable; PEAS, pancreatoenteric anastomotic stricture; SOD, sphincter of Oddi dysfunction.

**TABLE 2:** Technical and clinical success rates according to etiology.

	Etiology	*n*	Success cases	Rate, %	*p*‐value
Total technical success	PEAS	165	141	85.5	
	CP	104	87	83.7	0.8
	Others	30	26	86.7	
Total clinical success	PEAS	127	105	82.7	
	CP	32	26	81.3	1
	Others	18	17	94.4	

Abbreviations: CP, chronic pancreatitis; PEAS, pancreatoenteric anastomotic stricture.

**TABLE 3:** Adverse events according to onset time (<1 month and >1 month).

Procedure‐related AE (< 1 month)	*ｎ* = 57 (%)	Late AE (>1 month)	*ｎ* = 32 (%)
Pain	27 (47)	Stent migration	17 (53)
Pancreatic juice leakage‐related	12 (21)	Stent occlusion	13 (41)
Pancreatitis	6 (10)	Bleeding	2 (6)
Bleeding	5 (9)		
Stent migration	2 (3.5)		
Perforation	2 (3.5)		
Aneurysm and pseudocyst	1 (2)		
GW sharing	1 (2)		
Jejunal ulcer	1 (2)		

Abbreviation: AEs, adverse events; GW, guidewire.

### Tips for EUS‐PDD (initial EUS‐D/A)

EUS‐PDD techniques are reviewed elsewhere.^17^, ^18^ In a systematic review, the technical success rates of pancreatogram, EUS‐guided pancreatic duct cannulation, and EUS‐guided pancreatic duct stent placement were 87%, 79%, and 72%, respectively. EUS‐guided cannulation of a pancreatic duct has a lower success rate than a pancreatogram, and EUS‐guided placement of a pancreatic‐duct stent has a lower success rate than EUS‐guided cannulation of a pancreatic duct.^5^ The difficulties of EUS‐PDD include: (1) determination of the puncture point, (2) selection of a puncture needle and guidewire, (3) guidewire manipulation, and (4) dilation of the puncture route and stenting (Table 4). Proper technical procedures are important to increase the success rate and reduce the number of complications.

**TABLE 4:** Tips for initial endoscopic ultrasound‐drainage/anastomosis.

Steps	Difficult point	Technical tips	Figure
**1) Determination of the puncture point**			
**I) Distance**	Allow sufficient distance between the puncture	Positional relationship between the stricture and the vertebral is confirmed by CT and determines the point	Figure 2
	Point and the stricture (Figure 2‐i).	using the vertebral as an indicator.	
**II) Orient the EUS probe**	EUS probe orients the tail side, subsequent guidewire manipulation and device insertion is difficult.	Orient the EUS probe toward the stricture side with	Figure 2
		rotation under fluoroscopic guidance.	
**III) MPD configuration**	Steeply curved and tortuous MPD should be avoided because subsequent devices cannot be inserted.	“Re‐puncture” is strongly considered.	Video 1 and Figure 3
**2) Selection of the puncture needle and guidewire**			
**Needle**	Relationship between rigidity and puncture ability	Select the right needle and guidewire according to the patient and situation.	Table 5
**Guidewire**	Relationship between seeking and support ability		Table 6
**3) Guidewire manipulation**			
**I) Insertion into MPD**	Only contrast can be obtained unable to insert guidewire into the MPD.	“Moving scope technique”	Video 2
**II) Face the stricture side**	Guidewire inadvertently faces the tail side	“Loop technique”	NA
**III) Breaking through**	Breaking through the anastomosis/stricture	“Balloon occlusion technique”	Video 3
**4) Dilation of the puncture route and stenting**			
**Quickly and certainly**	Difficult to insert the dilation devices	Align with the axis by “Moving scope technique”	Video 4
	Scope instability in the dilation of stricture	“Double guidewire technique” to keep the stability	Video 1

Abbreviations: CT, computed tomography; EUS, endoscopic ultrasound; MPD, main pancreatic duct; NA, not applicable.

#### Determination of the puncture point

Determining the puncture position is crucial for tract dilation and completion of EUS‐PDD. We determine the puncture position by allowing a sufficient distance for stent placement between the puncture point and the stricture, orienting the EUS probe towards the stricture under fluoroscopic guidance, and selecting a point where the pancreatic duct is straight.

##### Allowing a sufficient distance between the puncture point and the stricture

**FIGURE 2:**
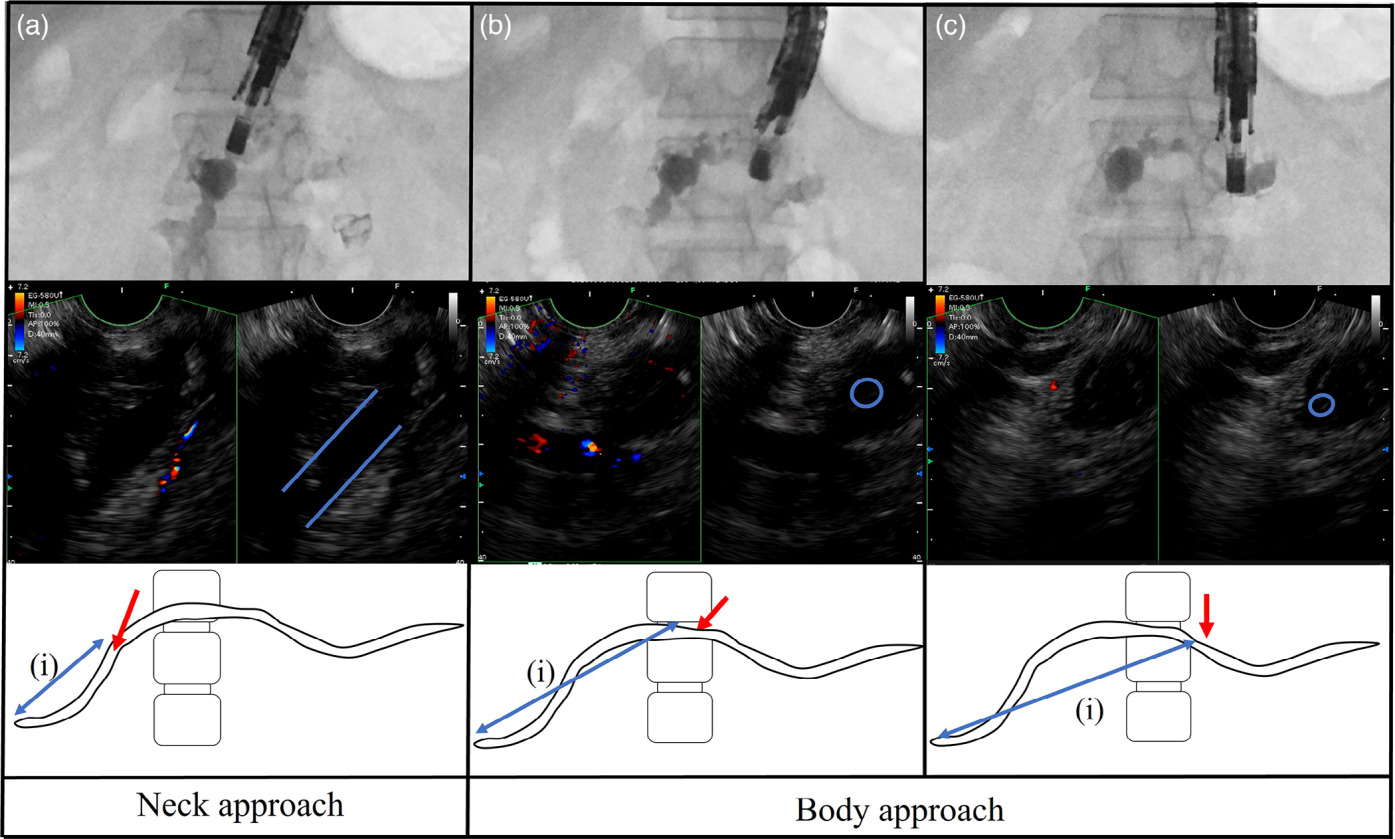
Relationship between scope position, endoscopic ultrasound (EUS) image, and schema. Main pancreatic duct is shown as a long axis on an EUS image (Figure 2a). Puncture from the pancreatic body provides sufficient distance from the papilla, but the pancreatic duct is observed in the short axis on the EUS image (Figure 2b). If the EUS probe faces the tail, guidewire manipulation and stenting are difficult (Figure 2c). The EUS probe should face the direction toward the stricture (Figure 2a,b) and tail side puncture as much as possible is better in consideration of the distance (i) for stent placement ((i); double arrow).

The selection of the puncture point should be based on the size of the pancreatic duct, the distance to the stricture, and the angle between the needle and the duct. Before the procedure, the configuration of the pancreatic duct should be evaluated by CT and/or MRCP. However, during the procedure, CT images are not linked to EUS images. Therefore, we typically establish a correlation between the vertebra and the stricture to assess the relationship. By doing so, we can select an appropriate puncture point based on the relationship between the vertebra and the EUS probe under fluoroscopic guidance (Figure 2). The puncture from the center of the vertebral (Figure 2a) is the neck approach and from the left side of the vertebral (Figure 2b) is the body approach in this case. When placing a pancreatic duct stent, it is important to ensure there is sufficient distance between the stricture and the puncture point (Figure 2(i)). This is particularly so in postoperative cases with a small residual pancreas. Therefore, it is advisable to select the tail side as much as possible for stent placement. If the puncture site and the stricture are too close to each other, “Re‐puncture” should be considered considering the subsequent stenting procedure.

##### Orienting the EUS probe towards the stricture under fluoroscopic guidance

The puncture point is important for determining whether guidewire induction to the stricture side is feasible during the procedure. This is particularly important in cases after pancreaticoduodenectomy, in which the EUS probe should face the direction of the stricture. However, the further towards the tail is the puncture point, the greater the movement of the EUS probe and guidewire towards the tail (Figure 2). In most cases, the scope should be rotated counterclockwise under fluoroscopic guidance to orient it towards the stricture side. The optimal point for EUS‐PDD access is typically between the gastric body and cardia.^19^

##### Selection of a straight section of the pancreatic duct for guidewire and device insertion

**FIGURE 3:**
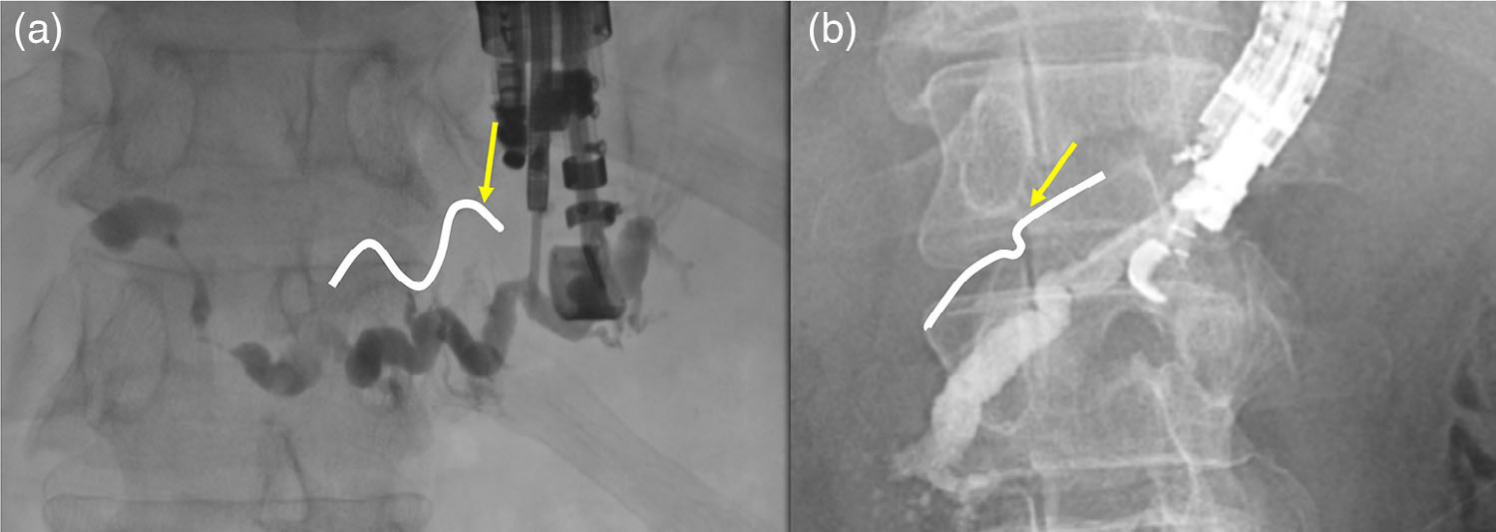
Difficult main pancreatic duct configuration for endoscopic ultrasound‐pancreatic duct drainage (schema with white line and puncture point with arrow). (a) Pancreato‐enteric anastomotic stricture after pancreatoduodenostomy with a tortuous main pancreatic duct. (b) In the case of pancreatic stones with endoscopic retrograde cholangiopancreatography failure, a puncture was performed at a curved region of the main pancreatic duct.

In some cases, the MPD may be tortuous in EUS. Even if MPD puncture and contrast can be performed, a curved pancreatic duct should be “Re‐punctured” before dilating it. A steeply curved and tortuous MPD should be avoided because devices cannot be inserted. We experienced a case in which dilation devices could not be inserted after guidewire insertion because the puncture was in a steeply curved and tortuous MPD (Figure 3), and “Re‐puncture” was easily performed. In Video 1, neither a balloon device (REN; Kaneka) nor a drill dilator (Tornus ES; Olympus Co.) could be inserted for 80 min after the initial puncture, but by re‐puncturing from the tail side, the puncture site was dilated in 5 min, the stricture was dilated in 15 min, and the procedure was completed to stent insertion in 25 min. The most difficult cases are those with a small remnant pancreatic volume after surgery and tortuous pancreatic ducts (Figure 3).

#### Selection of the puncture needle and guidewire

EUS‐PDD can be performed using 22‐, 21‐, 20‐, and 19‐gauge puncture needles, which are available with different tip shapes and materials. For EUS‐PDD, it is recommended to avoid Franseen or fork‐tip needles because they can tear the guidewire during manipulation. Needles with side holes should also be avoided.

Requirements for the needle for EUS‐PDD are as follows: (1) high puncture ability, (2) flexibility, and (3) keeping a straight shape (Table 5). We should puncture the thin duct in hard pancreatic parenchyma. Then, a very sharp tip needle is warranted. However, the tough needle is difficult to bend and difficult to target the thin duct. On the other hand, some flexible needle made from stainless steel has the nature of staying bent by scope angulation, and difficult to puncture the thin duct. The needle for EUS‐PDD should be selected according to the patient and situation. We prefer the EZ Shot 3 Plus (Olympus Medical Systems) with a coil sheath for EUS‐PDD. If a sharper needle is required because of very hard pancreatic parenchyma, I use the SonoTip ProControl (Medi‐Globe) stainless steel needle. In cases with a small pancreatic duct (median pancreatic duct diameter 3.5 mm, range 1–14 mm), a 22‐gauge needle can be used with a smaller (0.018‐ or 0.021‐inch) guidewire.^20^ However, it is challenging to manipulate small guidewires because they lack sufficient stiffness for dilation and stenting.

**TABLE 5:** Characteristics of puncture needles.

	EUS‐FNA needle				Access needle	
	EZ Shot 3 plus	Sono Tip	Expect	Flex 19	Echo‐Tip	Beacon
Puncture ability	○	◎	○	△	△	△
Flexibility	◎	◎	○	○	○	○
Keeping straight shape	◎	×	○	○	△	△
Guidewire introduction	△	△	△	△	△	◎
Contrast injection	○	○	○	○	○	×

For EUS‐PDD, the guidewire needs to be inserted into a thin pancreatic duct from the tip of the puncture needle and be able to penetrate the tortuous pancreatic duct with a tight stricture and support the insertion of devices through the hard pancreatic parenchyma (Table 6). In addition, guidewires for I‐EUS have a risk of guidewire sharing at the tip of the needle.

**TABLE 6:** Specifications of guidewires for endoscopic ultrasound‐guided pancreatic ductal drainage.

	Fielder	NovaGold	TracerMetro	VisiGlide2	EndoSelector	Jagwire
Diameter (inch)	0.018	0.018	0.021	0.025	0.025	0.025
Manuability	○	○	○	◎	◎	○
Torque ability	○	○	○	◎	◎	○
Support ability	○	△	△	○	○	○
Sharing risk	○	◎	○	◎	○	○

We routinely use a 19‐gauge needle with a sharp tip (EZ Shot 3 Plus) and a 0.025‐inch guidewire with a flexible tip (VisiGlide2; Olympus Medical Systems, Tokyo, Japan). We use the Selzinger method to puncture the pancreatic duct and align the needle tip with it. We typically use a half‐strength contrast medium (contrast mixed 1:1 with normal saline) to facilitate injection through the 19‐gauge needle. We confirm successful puncture by injecting contrast medium and inserting a 0.025‐inch guidewire as far as possible into the MPD, being careful to pass the stricture. Tables 5 and 6 summarize the desirable features of puncture needles and guidewires based on our experience.

#### Guidewire manipulation

It is important to maintain the scope position during guidewire manipulation so that the pancreatic duct is as close as possible to the ultrasound probe, and the guidewire is directed toward the stricture. If the stricture is overcome and the guidewire is guided towards the jejunum or duodenum, it will enhance scope stability and facilitate subsequent procedures. However, in cases of severe stricture, it may be necessary to place a stent to establish a connection between the pancreatic duct and the stomach, and then break through the stricture in a second session. Tips for guidewire manipulation will be provided for each situation.

##### Insertion into the MPD

Carefully confirm the tip of the needle on the ultrasound image because the guidewire often fails to penetrate the wall of the pancreatic duct. It is important to penetrate the wall using the Selzinger method and align the tip on the ultrasound screen while pulling the needle. If the angle between the pancreatic duct and the needle is perpendicular, it becomes difficult to insert the guidewire into the duct. Although contrast can be obtained, the guidewire may not reach the MPD. First, we change the puncture angle by manipulating the scope. In such cases, the guidewire can be inserted by gradually pushing the scope and turning the needle towards the stricture side (moving‐scope technique; Video 2).^21^ After guiding the wire to the stricture side, it is important to return to the puncture position.

##### Manipulation of the needle to the anastomosis/stricture side

If the guidewire is inadvertently directed towards the tail side, the needle may be removed for contrast only, but the situation can be rectified. Like the techniques used in EUS‐guided hepaticogastrostomy, the loop technique is used. This technique involves carefully pushing and turning the guidewire so that its soft part turns to the central side.^22^ If the loop technique is used, the needle is pulled into the pancreatic parenchyma, as in the bile duct. This prevents the guidewire from becoming trapped and enhances its torque performance.^23^

##### Breaking through the anastomosis/stricture

It is advised to refrain from executing a J‐turn with the guidewire prior to penetrating the stricture. Instead, it is recommended to gently navigate and explore with the tip without exerting force. If the scope lacks stability during the puncture, it can be stabilized by balloon dilation of the puncture site. Subsequently, the stricture can be traversed using a catheter and the maneuverability of the guidewire (balloon‐occlusion technique; Video 3). If it is challenging to traverse the pancreatic duct stricture/PEAS, we use a hydrophilic guidewire of 0.035‐inch diameter (Radifocus M, angled type; Terumo) to facilitate passage.

#### Dilation of the puncture route and stenting

Imoto et al. evaluated dilation instruments for the puncture route in EUS‐PDD.^24^ For EUS‐D/A, we employed two‐step dilation: initial dilation to insert devices and a second one with a balloon according to the type of T‐DAS. The initial dilation of the puncture route involved the utilization of a bougie dilator (ES Dilator; Zeon Medical) to make a space in the puncture tract, encompassing the pancreatic duct wall, pancreatic parenchyma, and gastric wall. If the bougie dilator fails to pass the puncture tract, we use an electric cautery dilator (6 Fr Cysto‐Gastro‐Set; Century Medical or Fine025; Medico's Hirata). To prevent the burning of the pancreas, non‐cautery‐assisted tract dilation can be attempted using rigid boogie dilators.^1^, ^4^, ^25^, ^26^ At our institution, the initial dilation approach involves the use of a bougie dilator.^27^, ^28^ In some cases, fully inserting a rigid dilator into the MPD is difficult because the puncture direction is almost perpendicular. We insert only the tip of the rigid boogie dilator in the MPD and subsequently insert a balloon catheter with a sharp tip (REN; Kaneka). This method is also useful for dilating the puncture tract. It is imperative to confirm that the shape of the endoscope matches that at the time of the initial puncture when inserting the dilation device (Video 4). In essence, adjusting the axis of the endoscope according to the devices and dilation point is of utmost importance. A recently introduced dilator known as Tornus ES (Olympus Co.) can be utilized.^29^ Tornus facilitates stoma dilation effortlessly through clockwise rotation. Subsequently, after dilation, a counterclockwise rotation of Tornus ES is necessary for removal. This drill dilator significantly enhances the safety and ease of conducting EUS‐PDD (Video 1).

**FIGURE 4:**
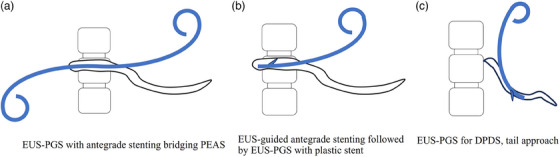
Stent placement in endoscopic ultrasound‐guided pancreatogastrostomy (EUS‐PGS). (a) EUS‐PGS with antegrade stenting bridging pancreato‐enteric anastomosis (PEAS). The tip of the trans‐luminal drainage/anastomotic stent is placed in the jejunum. (b) In EUS‐guided antegrade stenting followed by EUS‐PGS with a plastic stent, trans‐luminal drainage/anastomotic stent was placed in the main pancreatic duct. (c) EUS‐PGS for duct disconnecting syndrome (DPDS) by the tail approach. Cases post‐Whipple resection with small remnant pancreas are available as well.

EUS‐PDD requires adequate dilation of the puncture tract to facilitate the insertion of a T‐DAS deep into the MPD, thereby preventing stent displacement.^4^, ^26^ Variation in this technique depends on the puncture site and whether the stent will be placed transmurally or in an antegrade manner across an anastomosis or papilla. At our institution, we simultaneously create a pancreaticogastrostomy and pancreaticoenterostomy by passing the distal end of the double pigtail plastic stent through the papilla or anastomosis into the intestine, while deploying the proximal end into the gastric lumen (Figure 4a).^26^, ^30^ We perform the double‐guidewire technique using a double‐lumen catheter (Uneven Double Lumen Cannula; Piolax Medical) and a 0.035‐inch angled guidewire (Revowave Hard and Ultrahard; Piolax Medical) to stabilize the scope and position the safety wire. Subsequently, we position a 7 Fr double‐pigtail plastic stent (Zimmon; Cook Medical or Through‐Pass DP; Gadelius Medical) spanning the pancreatic duct stricture/PEAS and extending through the MPD into the stomach. If the stricture is too severe to traverse, the distal end of the stent should be positioned in the MPD using the TYPE‐IT stent (Gadelius Medical, Tokyo, Japan), which has a tapered tip and a large tail on the gastric side (Figure 4b).^31^ The stent should preferably be equipped with a locking system for the pulling back function. The endoscopist needs advanced EUS skills and much experience in stent placement. If a sufficient distance between the stricture and the puncture point cannot be obtained, such as in DPDS, a stent is placed at the tail side similar to EUS‐PGS for DPDS, that is a tail approach (Figure 4c).

### EUS system

It is important to use an I‐EUS‐specific scope. A convex echoendoscope and a universal‐type ultrasound processor (EG‐740UT and SU‐1; Fujifilm) are used for I‐EUS at our institution. Compared to the EG‐580UT, the new EG‐740UT has a shorter rigid part at the tip of the scope to reduce the length at up‐angles, thereby improving the maneuverability of the scope tip. In addition, the placement of the endoscopic camera behind the elevator has improved visibility and prevents stent misplacement. In addition, the ultrasound image at the puncture point has fewer blind areas, preventing a double puncture of the digestive tract wall and blood vessels. The scope is equipped with a guidewire‐locking function, which makes it suitable for I‐EUS. This also facilitates device exchange and reduces pancreas juice leakage by shortening the procedure duration. In addition, the scope channel is large enough to allow simultaneous insertion of a duodenal stent in cases in which I‐EUS is necessary because of duodenal stenosis caused by a tumor.

## SUMMARY

In conclusion, to overcome a stricture and deploy a stent, it is advisable to: (1) determine the puncture point, (2) select an appropriate puncture needle and guidewire, (3) manipulate the guidewire, and (4) dilate the puncture route to reduce serious complications. If the desired distance and direction cannot be achieved by pancreatography or if the guidewire or dilator fails to lead towards the stricture, “Re‐puncture” should be considered to facilitate subsequent dilation and stenting.

## CONFLICT OF INTEREST STATEMENT

Hiroyuki Isayama is supported by honoraria from the FUJIFILM Corporation, Tokyo, Japan and FUJIFILM Healthcare Corporation, Tokyo, Japan. The other authors declare no conflict of interest.

## Supporting information


**VIDEO S1** Steeply curved and tortuous MPD observed by EUS.
A balloon device, drill dilator, or boogie dilator could be inserted into the MPD. Eighty minutes passed without the insertion of dilation devices. Following “Re‐puncture” from the tail side, the procedure was completed in 25 min. Even if MPD puncture and contrast can be performed, if the pancreatic duct is curved, it is strongly recommended to perform a “Re‐puncture” before dilating the pancreatic duct.

**VIDEO S2** Moving‐scope technique (For guidewire).
If the guidewire cannot be inserted into the MPD because the puncture site is perpendicular to the MPD, the EUS probe should face the stricture site to facilitate the insertion of the guidewire.

**VIDEO S3** Balloon dilation technique.
The stricture can be traversed using the maneuverability of the guidewire by dilating the balloon at the puncture site.

**VIDEO S4** Moving‐scope technique (For dilation device).
The shape of the scope returns to that at the time of puncture on fluoroscopic and ultrasonographic imaging.

## References

[1] Kurihara T , Itoi T , Sofuni A , Itokawa F , Moriyasu F . Endoscopic ultrasonography‐guided pancreatic duct drainage after failed endoscopic retrograde cholangiopancreatography in patients with malignant and benign pancreatic duct obstructions. Dig Endosc 2013; 25 (Suppl 2): 109–116. 23617660 10.1111/den.12100

[2] Will U , Reichel A , Fueldner F , Meyer F . Endoscopic ultrasonography‐guided drainage for patients with symptomatic obstruction and enlargement of the pancreatic duct. World J Gastroenterol 2015; 21: 13140–13151. 26674313 10.3748/wjg.v21.i46.13140 PMC4674733

[3] Krafft MR , Nasr JY . Anterograde endoscopic ultrasound‐guided pancreatic duct drainage: A technical review. Dig Dis Sci 2019; 64: 1770–1781. 30734236 10.1007/s10620-019-05495-9

[4] Fujii LL , Topazian MD , Abu Dayyeh BK *et al.* EUS‐guided pancreatic duct intervention: Outcomes of a single tertiary‐care referral center experience. Gastrointest Endosc 2013; 78: 854–864.e1. 23891418 10.1016/j.gie.2013.05.016

[5] Basiliya K , Veldhuijzen G , Gerges C *et al.* Endoscopic retrograde pancreatography‐guided versus endoscopic ultrasound‐guided technique for pancreatic duct cannulation in patients with pancreaticojejunostomy stenosis: A systematic literature review. Endoscopy 2021; 53: 266–276. 32544958 10.1055/a-1200-0199

[6] Bataille L , Deprez P . A new application for therapeutic EUS: Main pancreatic duct drainage with a “pancreatic rendezvous technique”. Gastrointest Endosc 2002; 55: 740–743. 11979263 10.1067/mge.2002.123621

[7] Nakai Y , Kogure H , Isayama H , Koike K . Endoscopic ultrasound‐guided pancreatic duct drainage. Saudi J Gastroenterol 2019; 25: 210–217. 30632484 10.4103/sjg.SJG_474_18 PMC6714474

[8] Tyberg A , Bodiwala V , Kedia P *et al.* EUS‐guided pancreatic drainage: A steep learning curve. Endosc Ultrasound 2020; 9: 175–179. 32584312 10.4103/eus.eus_3_20 PMC7430898

[9] Ahmed Ali U , Pahlplatz JM , Nealon WH , van Goor H , Gooszen HG , Boermeester MA . Endoscopic or surgical intervention for painful obstructive chronic pancreatitis. Cochrane Database Syst Rev 2015; 2015: Cd007884. 25790326 10.1002/14651858.CD007884.pub3 PMC10710281

[10] Brauer BC , Chen YK , Fukami N , Shah RJ . Single‐operator EUS‐guided cholangiopancreatography for difficult pancreaticobiliary access (with video). Gastrointest Endosc 2009; 70: 471–479. 19560768 10.1016/j.gie.2008.12.233

[11] van der Merwe SW , van Wanrooij RLJ , Bronswijk M *et al.* Therapeutic endoscopic ultrasound: European Society of Gastrointestinal Endoscopy (ESGE) Guideline. Endoscopy 2022; 54: 185–205. 34937098 10.1055/a-1717-1391

[12] Chahal P , Baron TH , Topazian MD , Petersen BT , Levy MJ , Gostout CJ . Endoscopic retrograde cholangiopancreatography in post‐Whipple patients. Endoscopy 2006; 38: 1241–1245. 17163326 10.1055/s-2006-945003

[13] Kogure H , Sato T , Nakai Y *et al.* Endoscopic management of pancreatic diseases in patients with surgically altered anatomy: Clinical outcomes of combination of double‐balloon endoscopy‐ and endoscopic ultrasound‐guided interventions. Dig Endosc 2021; 33: 441–450. 32434287 10.1111/den.13746

[14] Suzuki A , Ishii S , Fujisawa T *et al.* Efficacy and safety of peroral pancreatoscopy through the fistula created by endoscopic ultrasound‐guided pancreaticogastrostomy. Pancreas 2022; 51: 228–233. 35584379 10.1097/MPA.0000000000002003

[15] Takahashi S , Tomishima K , Takasaki Y *et al.* Successful retrieval of a migrated stent in the pancreatic duct after endoscopic ultrasound‐guided pancreaticogastrostomy with peroral pancreatoscopy. Endoscopy 2024; 56: E136–E137. 38359879 10.1055/a-2239-4737 PMC10869217

[16] Kiyanagi A , Fujisawa T , Ishii S *et al.* Usefulness of routine plain CT the day after an interventional EUS procedure. Saudi J Gastroenterol 2021; 27: 275–282. 34380872 10.4103/sjg.sjg_81_21 PMC8555768

[17] Itoi T , Kasuya K , Sofuni A *et al.* Endoscopic ultrasonography‐guided pancreatic duct access: Techniques and literature review of pancreatography, transmural drainage and rendezvous techniques. Dig Endosc 2013; 25: 241–252. 23490022 10.1111/den.12048

[18] Itoi T , Yasuda I , Kurihara T , Itokawa F , Kasuya K . Technique of endoscopic ultrasonography‐guided pancreatic duct intervention (with videos). J Hepatobiliary Pancreat Sci 2014; 21: E4–E9. 24123911 10.1002/jhbp.43

[19] Siddiqui UD , Levy MJ . EUS‐guided transluminal interventions. Gastroenterology 2018; 154: 1911–1924. 29458153 10.1053/j.gastro.2017.12.046

[20] Matsunami Y , Itoi T , Sofuni A *et al.* Evaluation of a new stent for EUS‐guided pancreatic duct drainage: Long‐term follow‐up outcome. Endosc Int Open 2018; 6: E505–E512. 29713675 10.1055/s-0044-101753 PMC5906111

[21] Ueno S , Ogura T , Higuchi K . Moving scope technique for guidewire insertion during endoscopic ultrasound‐guided hepaticogastrostomy. Dig Endosc 2021; 33: e109–e110. 33970508 10.1111/den.13993

[22] Matsubara S , Nakagawa K , Suda K , Otsuka T , Oka M , Nagoshi S . Practical tips for safe and successful endoscopic ultrasound‐guided hepaticogastrostomy: A state‐of‐the‐art technical review. J Clin Med 2022; 11: 1591. 35329917 10.3390/jcm11061591 PMC8949311

[23] Ogura T , Masuda D , Takeuchi T , Fukunishi S , Higuchi K . Liver impaction technique to prevent shearing of the guidewire during endoscopic ultrasound‐guided hepaticogastrostomy. Endoscopy 2015; 47: E583–E584. 26649471 10.1055/s-0034-1393381

[24] Imoto A , Ogura T , Higuchi K . Endoscopic ultrasound‐guided pancreatic duct drainage: Techniques and literature review of transmural stenting. Clin Endosc 2020; 53: 525–534. 32967409 10.5946/ce.2020.173 PMC7548157

[25] Honjo M , Itoi T , Tsuchiya T *et al.* Safety and efficacy of ultra‐tapered mechanical dilator for EUS‐guided hepaticogastrostomy and pancreatic duct drainage compared with electrocautery dilator (with video). Endosc Ultrasound 2018; 7: 376–382. 29882518 10.4103/eus.eus_2_18 PMC6289009

[26] Krafft MR , Croglio MP , James TW , Baron TH , Nasr JY . Endoscopic endgame for obstructive pancreatopathy: Outcomes of anterograde EUS‐guided pancreatic duct drainage. A dual‐center study. Gastrointest Endosc 2020; 92: 1055–1066. 32376334 10.1016/j.gie.2020.04.061

[27] Amano M , Ogura T , Onda S *et al.* Prospective clinical study of endoscopic ultrasound‐guided biliary drainage using novel balloon catheter (with video). J Gastroenterol Hepatol 2017; 32: 716–720. 27420770 10.1111/jgh.13489

[28] Kanno Y , Ito K , Koshita S *et al.* Efficacy of a newly developed dilator for endoscopic ultrasound‐guided biliary drainage. World J Gastrointest Endosc 2017; 9: 304–309. 28744342 10.4253/wjge.v9.i7.304 PMC5507821

[29] Hara K , Okuno N , Haba S *et al.* Utility of a novel drill dilator for easier EUS‐guided pancreatic duct drainage. J Hepatobiliary Pancreat Sci 2022; 29: e91–e92. 35243809 10.1002/jhbp.1130

[30] Tyberg A , Sharaiha RZ , Kedia P *et al.* EUS‐guided pancreatic drainage for pancreatic strictures after failed ERCP: A multicenter international collaborative study. Gastrointest Endosc 2017; 85: 164–169. 27460387 10.1016/j.gie.2016.07.030

[31] Itoi T , Sofuni A , Tsuchiya T *et al.* Initial evaluation of a new plastic pancreatic duct stent for endoscopic ultrasonography‐guided placement. Endoscopy 2015; 47: 462–465. 25590174 10.1055/s-0034-1391083

